# SUMO-specific Isopeptidases Tuning Cardiac SUMOylation in Health and Disease

**DOI:** 10.3389/fmolb.2021.786136

**Published:** 2021-11-19

**Authors:** Paul W. Hotz, Stefan Müller, Luca Mendler

**Affiliations:** Institute of Biochemistry II, Gustav Embden Zentrum, Faculty of Medicine, Goethe University Frankfurt, Frankfurt, Germany

**Keywords:** SUMO, SENP, heart failure, heart, ischemia reperfusion (I/R) injury

## Abstract

SUMOylation is a transient posttranslational modification with small-ubiquitin like modifiers (SUMO1, SUMO2 and SUMO3) covalently attached to their target-proteins via a multi-step enzymatic cascade. SUMOylation modifies protein-protein interactions, enzymatic-activity or chromatin binding in a multitude of key cellular processes, acting as a highly dynamic molecular switch. To guarantee the rapid kinetics, SUMO target-proteins are kept in a tightly controlled equilibrium of SUMOylation and deSUMOylation. DeSUMOylation is maintained by the SUMO-specific proteases, predominantly of the SENP family. SENP1 and SENP2 represent family members tuning SUMOylation status of all three SUMO isoforms, while SENP3 and SENP5 are dedicated to detach mainly SUMO2/3 from its substrates. SENP6 and SENP7 cleave polySUMO2/3 chains thereby countering the SUMO-targeted-Ubiquitin-Ligase (StUbL) pathway. Several biochemical studies pinpoint towards the SENPs as critical enzymes to control balanced SUMOylation/deSUMOylation in cardiovascular health and disease. This study aims to review the current knowledge about the SUMO-specific proteases in the heart and provides an integrated view of cardiac functions of the deSUMOylating enzymes under physiological and pathological conditions.

## Introduction

Cardiovascular diseases such as atherosclerosis, acute coronary disease, cardiomyopathies and heart failure are leading causes of morbidity and mortality worldwide (Murray and Lopez, 1997). Much progress has been made to identify the underlying molecular mechanisms associated with cardiac diseases, nevertheless, only few new drugs were derived from such efforts within the past years (Kairouz et al., 2012; Münzel et al., 2015; Mendler et al., 2016; Heusch, 2020). Accordingly, there is an urgent need to deeper understand the molecular mechanisms of cardiac pathologies to develop new therapeutic approaches for effective cardioprotection.

**FIGURE 1:**
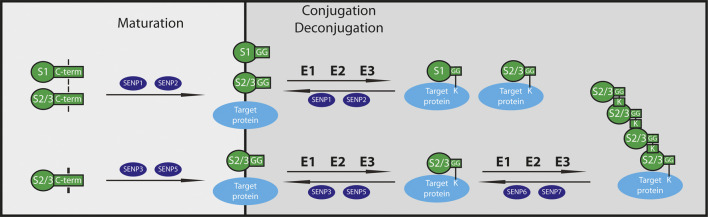
Function of SUMO/Sentrin specific isopeptidases. SUMO isoforms are synthetized as precursor proteins and cleaved by SUMO-specific isopeptidases (SENPs). During maturation, SENP1 and SENP2 process both SUMO1 and SUMO2/3, whereas SENP3 and SENP5 hydrolyse only SUMO2/3. Mature SUMO proteins get conjugated to target proteins through an enzymatic cascade similar to ubiquitination catalyzed by E1, E2 and E3 enzymes in order to form mono- or polySUMOylated substrates. To maintain the equilibrium of SUMOylation and deSUMOylation, SENPs play a crucial role as deconjugases. SENP1 and SENP2 detach SUMO1 and SUMO2/3 from monoSUMOylated substrates, while the SUMO2-specific isopeptidases SENP3 and SENP5 remove SUMO2/3 from targets. SENP6 and SENP7 cut mainly SUMO2/3 from polySUMO2/3-chains of target proteins. S1: SUMO1, S2/3: SUMO2/3, G: Glycine, K: Lysine.

Post-translational modifications (PTM), such as phosphorylation, acetylation or methylation represent a powerful tool to alter protein activities and their cellular localization and function in living organisms. Small proteins like ubiquitin and ubiquitin-like modifiers (ULM) can become covalently attached to distinct lysine residues of their target proteins regulating several aspects of cellular proteostasis. SUMO (small ubiquitin-related modifier) is a typical ULM (van der Veen and Ploegh, 2012; Flotho and Melchior, 2013) with four different isoforms in mammals (SUMO1-4), where only SUMO1-3 are expressed ubiquitously. SUMO1 shares about ∼50% sequence identity with SUMO2 and SUMO3, which are ∼97% identical in their sequence and therefore referred to as SUMO2/3. SUMO1-3 are synthesized as precursor proteins that undergo carboxy-terminal processing (Müller et al., 2001) to expose a di-glycine (diGly) motif required for conjugation. This cleavage is performed by cysteine proteases of the sentrin-specific protease (SENP) family (Hickey et al., 2012; Nayak and Müller, 2014; Kunz et al., 2018) (Figure 1). SUMO4 lacks a critical residue in the SENP recognition site (Owerbach et al., 2005), likely rendering it resistant to processing and conjugation. The conjugation of mature SUMO is an ATP-dependent process involving an enzymatic cascade comprised of a single dimeric E1 activating enzyme (SAE1/UBA2), a sole E2 conjugating enzyme (UBE2I or UBC9) and a limited number of facultative E3 Ligases such as the PIAS (protein inhibitors of activated STATs) family, RanBP2 and others (Figure 1). Processed SUMO is conjugated via its C-terminal diGly motif to one or more lysine residues of target proteins generating mono- or multiSUMOylated proteins. SUMO, in particular SUMO2/3, can also form polymeric chains (polySUMOylation) by attachment to internal lysine residues of SUMO. In general, SUMO modifies hundreds of cellular proteins and is thereby able to alter protein-protein interaction networks, protein activities or subcellular distribution of proteins. SUMO mediated protein-protein interaction occurs via SUMO-interaction motifs (SIMs), with SUMOylated and SIM-containing proteins binding non-covalently to each other (Kerscher, 2007; Gareau and Lima, 2010). Due to fast changes in the cellular environment a highly dynamic mechanism is needed to change protein properties accordingly. Therefore, the distinct SUMO state of a protein is tightly controlled by the balance of conjugation and deconjugation. Members of the SENP family represent the best characterized SUMO deconjugating enzymes (Figure 1). Other deSUMOylating enzymes have emerged in the past years i.e. Desi-1/2 with restricted substrate specificity, and USPL1 important for small ribonucleic particle assembly and pre-mRNA splicing in Cajal bodies; however, very limited information is available about their function so far (Gillies and Hochstrasser, 2012; Schulz et al., 2012; Shin et al., 2012; Hutten et al., 2014; Nayak and Müller, 2014). In humans, six different deSUMOylating SENPs (SENP1-3, SENP5-7) are known which all share a conserved catalytic centre, consisting of a typical cysteine protease catalytic triad (Cys-His-Asp) (Mukhopadhyay and Dasso, 2007; Hickey et al., 2012). The six mammalian SENP enzymes originate evolutionary from two different branches, designated Ulp1 and Ulp2 referring to the assignment of the related yeast enzymes. SENP1-5 are related to the Ulp1 family, whereas Ulp2 is close to the SUMO chain editing enzymes SENP6 and SENP7 (Hickey et al., 2012; Nayak and Müller, 2014). SENP1 and SENP2 localize in nucleus, nuclear envelope and cytoplasm and are active in the maturation and deconjugation of all three SUMO isoforms (Hang and Dasso, 2002; Reverter and Lima, 2004; Shen et al., 2006; Kolli et al., 2010; Goeres et al., 2011), while SENP3 and SENP5 localize mainly in the nucleus (with nucleolar dominance) favouring processing and removal of SUMO2/3 from modified proteins (Nishida et al., 2000; Gong and Yeh, 2006; Zunino et al., 2007; Haindl et al., 2008; Zunino et al., 2009; Kolli et al., 2010) (Figure 1). SENP6 and SENP7 are preferentially trimming SUMO chains and are found predominantly in the nucleoplasm with a smaller fraction of SENP7 expressed in the cytoplasm as well (Lima and Reverter, 2008; Shen et al., 2009; Kolli et al., 2010). Several biochemical studies pinpoint towards the SENPs as critical enzymes to control balanced SUMOylation/deSUMOylation in cardiovascular health and disease. In this review, we provide an integrative view about the role of SENP enzymes in the cardiovascular system and critically discuss their cardiac-specific functions.

## SENPs in Developing Heart

Several lines of evidence suggest that the SUMO-conjugation/deconjugation system plays an essential role during embryonic development. The comparative analysis of SUMO1, SUMO2 and SUMO3 knockout mouse mutants revealed embryonic lethality upon deletion of SUMO1 or SUMO2 genes, whereas SUMO3 was dispensable for development (Wang et al., 2011; Wang et al., 2014). On the other hand, germ-line deletion of SENP1, SENP2 or SENP3 genes in mice showed embryonic lethality as well (Cheng et al., 2007; Kang et al., 2010; Lao et al., 2018; Yu et al., 2020). Among them, SUMO1 and SENP2 seem to play the most significant role in keeping the balance of conjugation/deconjugation during cardiac development as knockout mice developed congenital heart defects (atrial/ventral septum defects) in both cases (Kang et al., 2010; Wang et al., 2011; Fu et al., 2014). Although several experimental data support this idea, other research groups questioned the cardiac-specific role of SUMO1 claiming that the loss of SUMO1 is compensated by SUMO2- and -3 during development (Evdokimov et al., 2008; Zhang et al., 2008). Moreover, the placental insufficiency of the SENP2 germline mutant mice complicated the assessment of the cardiac phenotype as cardiac abnormalities arise at stages when embryogenesis greatly depends on placental function (Chiu et al., 2008). Indeed, epiblast-specific conditional SENP2 mouse mutants (with a healthy placenta containing intact SENP2) did not exhibit any heart defects, demonstrating that the heart abnormalities of SENP2 global knockout are likely not primary, but secondary defects due to placental deficiencies (Fu et al., 2014; Maruyama et al., 2016). Altogether, given the general and multi-organ role of SENPs during embryonic development, cardiac-specific deletion of single or multiple SENPs instead of germ-line knockout would be essential to delineate their specific role in cardiac development. Nevertheless, among the SENP family members, SENP2 was recently identified as the predominant form being upregulated during postnatal heart development (Chen et al., 2021). Remarkably, SENP2 protein expression followed the postnatal upregulation of the transcript levels and revealed significantly higher levels in isolated murine cardiomyocytes (CM) than in cardiac fibroblasts (Chen et al., 2021). Regarding the expression of other SENPs, only limited and controversial data are available during embryonic and postnatal heart development with lower expression of SENP1, SENP6 and SENP7 in postnatal heart, but with variable - increasing (Zhang et al., 2020) or decreasing (Chen et al., 2021) - protein levels of SENP3 and SENP5. Notably, detection of SENP proteins from murine heart is challenging since most of the commercially available antibodies give only faint or multiple signals. As no proteome data regarding SENPs are available in developing heart, it is unclear whether the low cardiac protein expression of SENPs complicates their detection as well.

## SENPs in Adult Heart

### Healthy Heart

**FIGURE 2:**
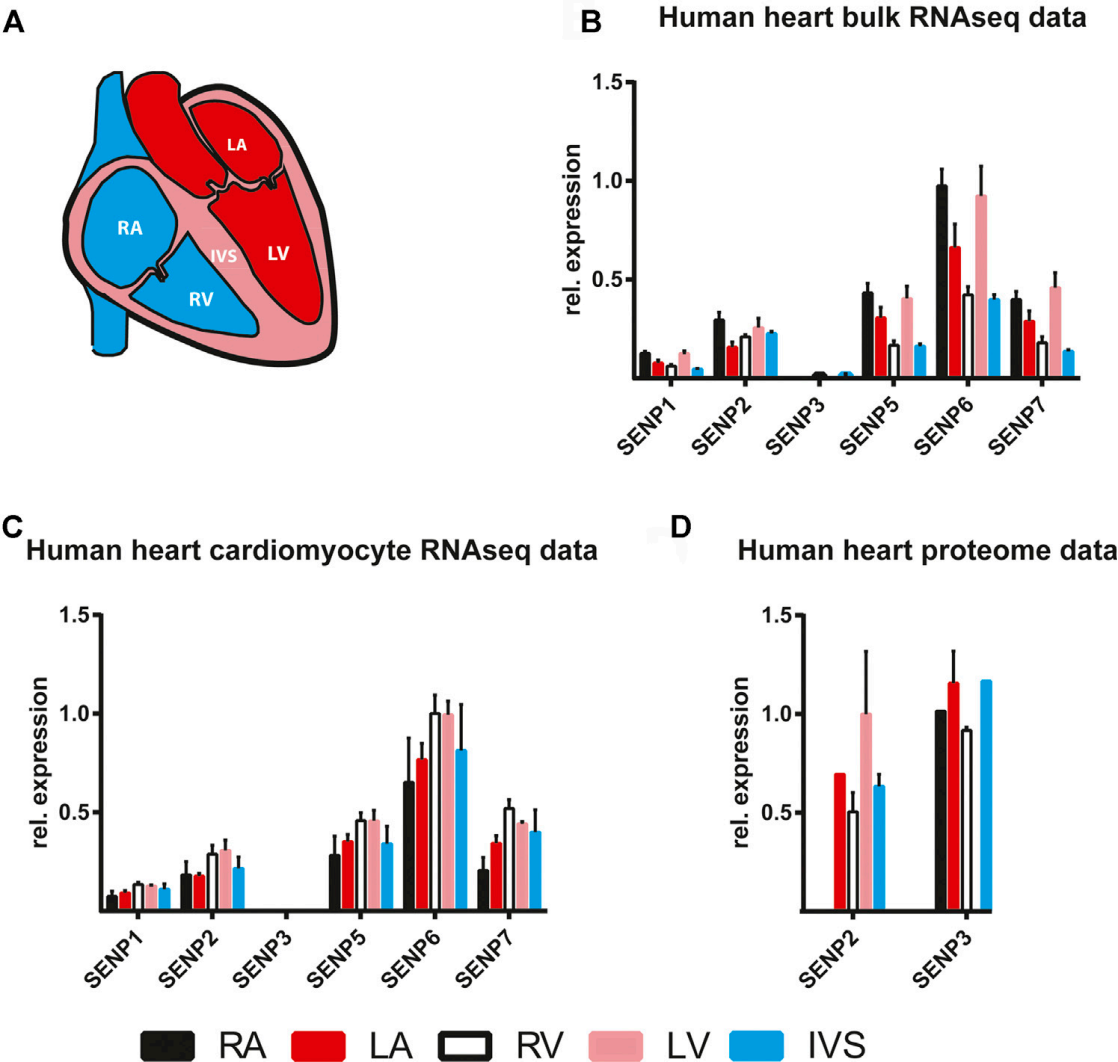
RNA and protein expression of SENPs in different anatomical areas of the human heart **(A)** Graphical illustration of the anatomical regions of the human heart **(B, C)** Non-failing human heart samples, declined for transplantation were used for either bulk RNA isolation and sequencing (B, (Johnson et al., 2018), or for single cell RNA analysis (C (Litviňuková et al., 2020)). By normalizing the original SENP expression data to those of the cardiac housekeeping gene GAPDH in both studies, region-specific SENP expression in the human heart has been re-analyzed and compared **(D)** SENP protein expression in human heart regions (Doll et al., 2017). N = one to three, mean ± SD are shown. RA: Right atrium, LA: Left atrium, RV: right ventricle, LV: left ventricle, IVS: interventricular septum.

The availability of RNA sequencing and proteomic datasets from adult human healthy heart samples allowed us to assess differential expression of the deSUMOylation machinery in the adult heart. Johnson et al. performed region-specific bulk RNA sequencing in human hearts, declined for transplantation, while Litviňuková et al. (2020) accomplished single cell and single nuclei RNA sequencing analysis in a cell-type specific manner. We have re-analyzed both datasets (Figure 2) and compared the bulk vs cardiomyocyte specific RNAseq data (Figure 2B,C) in different anatomical (atrial, ventricular, and septal) areas of the human heart (Figure 2A, Li et al., 2017). RNASeq data revealed remarkably similar expression pattern in both datasets with higher relative expression of SENP6, SENP5 and SENP7 and lower expression of SENP1, SENP2 and SENP3 in most anatomical regions (Figure 2B, C). Notably, bulk RNA sequencing displayed greater regional differences with higher SENP expression in right atrium and left ventricle, possibly reflecting the distinct cell-type composition (cardiomyocytes, fibroblasts, endothelial cells, macrophages etc) of the respective cardiac region (Johnson et al., 2018; Litviňuková et al., 2020). In line with this idea, RNA sequencing data from cardiomyocytes showed higher SENP RNA levels mainly in ventricular cardiomyocytes (Figure 2C). Similarly, Tucker et al. (2020) described the highest SENP RNA levels in a specific cluster of ventricular cardiomyocytes. Notably, however, high SENP expression was as well detected in some types of fibroblasts and endothelial cells enriched in right atrium. Thus, transcriptional regulation seems to follow a cell-type specific manner in the heart with significant differences between distinct SENPs. Strikingly, SENP3 expression was almost below the detection limit in all cardiac regions whereas SENP6 revealed the highest RNA expression in all datasets (Figure 2B, C, and Tucker et al., 2020). The low relative expression of SENP3 mRNA is surprising when considering proteomic data obtained from healthy human hearts, where exclusively SENP3 and SENP2 proteins were detectable (Figure 2D, Doll et al., 2017). These data are in accordance with the findings in mouse heart discussed above (Chen et al., 2021), where SENP2 protein was upregulated during postnatal development in adult mouse heart, whereas other SENPs seemed to be more dynamically regulated. Remarkably, SENP6 protein was only present at low level in right atrium whereas no other SENP was detectable in any cardiac region (Doll et al., 2017). Although technical issues can not be ruled out when a given SENP protein was not identified in proteome analysis, the obvious discrepancy between transcript and protein levels - e.g. low transcript and high protein levels of SENP3, whereas high transcript and low protein levels of SENP6 - points toward a significant posttranscriptional regulation of distinct SENPs in the human heart. Indeed, protein stability of the redox-sensitive enzyme SENP3 has recently been shown to undergo ROS dependent dynamic regulation in oxidative stress, hypoxia or in vascular remodeling (Yan et al., 2010; Guo et al., 2013; Cai et al., 2021). However, altered transcript or protein levels of SENPs do not necessarily reflect changes in activity, since enzyme activity can be uncoupled from protein expression as shown by SENP3 inactivation under hypoxic condition (Kunz et al., 2016). Therefore, SENP activity-based assays are not only essential *in vitro* or in cell culture systems but also *in vivo* in mouse or human tissues (Kunz et al., 2019; Hotz et al., 2020). By applying fluorescence-based- or haemagglutinin (HA)- coupled SUMO1 or SUMO2/3-probes we confirmed that SUMO2/3-specific SENPs are more active in wild-type mouse hearts than the SUMO1-specific ones, with SENP3 showing the highest activity among them (Kunz et al., 2019; Hotz et al., 2020). Altogether, SENP2 and SENP3 seem to play a significant role in maintaining the balance of SUMO-dependent cellular homeostasis of the adult human heart, however, several important pieces of information are still missing regarding SENP functions under physiological and pathological conditions.

### Heart Failure and Cardiomyopathies

The heart adapts to several stimuli and utilizes different mechanisms coping against stress. However, pathological adaptation of the heart leads to cardiomyopathies, cardiac dysfunction and ultimately to heart failure. Exploring the underlying mechanisms and identifying responsible factors is crucial to progress towards better treatment strategies.

Data from animal models and human patients pinpoint towards balanced SUMO conjugation and deconjugation as a key factor for cardiac stress adaption (Kho et al., 2011; Gupta et al., 2014; Maejima and Sadoshima, 2014; Kim et al., 2015b). In a pressure overload induced model of cardiac hypertrophy and heart failure, elevated SUMO1 conjugation suppresses the hypertrophic phenotype and inhibits the hypertrophic responses in cultivated cardiomyocytes (Lee et al., 2014). In contrast, constitutive heart specific SUMO2 overexpression induces dose-dependent hypertrophy and cardiomyopathy via inhibiting calpastatin and thereby activating calpain 2, an important member of the cellular proteolytic system (Kim et al., 2015b). The different outcome of the enforced SUMO1 or SUMO2 expression in the heart is very likely the consequence of distinct subsets of target proteins engaged by either SUMO1 or SUMO2. Recently, great efforts have been made to define and understand the cardiac SUMOylome (Hendriks et al., 2018; Hotz et al., 2020), however, the full range of SUMO targets remains to be uncovered in the heart. Since SUMO deconjugases greatly contribute to dynamic changes of the SUMOylome, altered function of SENPs are expected to lead to heart pathologies. Yet, it is not an easy task to uncover the cardiac specific role of SENPs in adult hearts given that SENP1, SENP2 and SENP3 germline mutants are embryonic lethal. Studies analyzing conditional knockout/knockdown of SENP2 or overexpression of SENP1, SENP2, and SENP5 in adult hearts have been published (Kim et al., 2012; Kim et al., 2015a; Cai et al., 2015; Hendriks et al., 2018; Hotz et al., 2020), however, no data exist in the literature regarding the cardiac specific conditional mutants of SENP3, SENP6 and SENP7.

**FIGURE 3:**
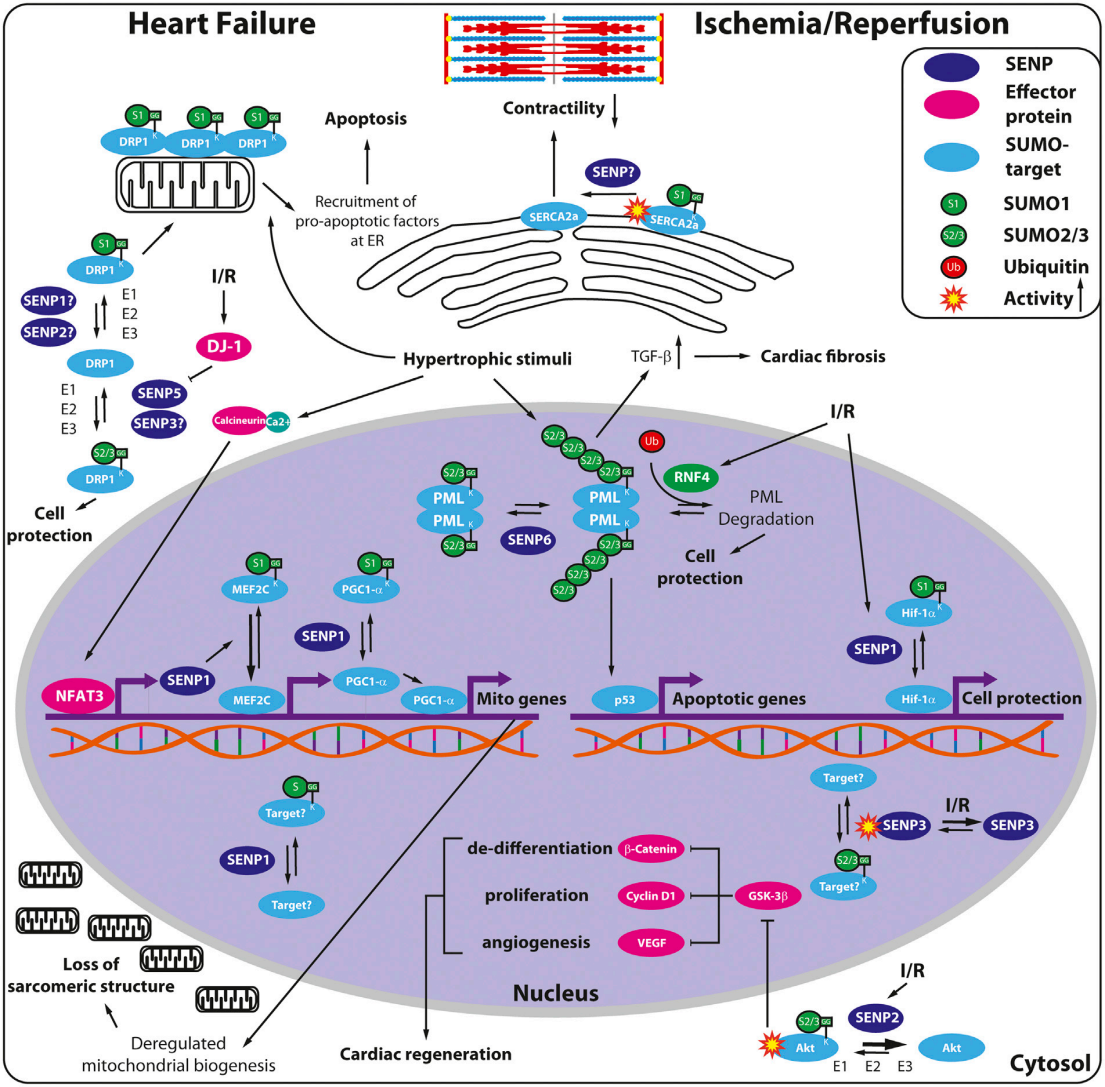
Overview of SENP functions in heart failure and cardiac ischemia-reperfusion injury. Hypertrophic stimuli induce SENP1 in a calcineurin/NFAT3-dependent manner in the failing heart. SENP1 deSUMOylates and thereby activates the MADS-box transcription factor MEF2C facilitating PGC1-α expression, whereas the transcriptional activity of PGC1-α is directly enhanced by SENP1. Hyperactivation of PGC1-α leads in turn to deregulated mitochondrial biogenesis and loss of the sarcomeric structure. Moreover, decreased SUMOylation of SERCA2a reduces enzyme stability and activity thereby worsening cardiac contractility in heart failure. Hypertrophic stimuli, by regulating various SENPs, may also change the balance between SUMO1- and SUMO2/3-modified DRP1, with SUMO1-DRP1 oligomerizing on the outer mitochondrial membranes thereby creating an ER-mitochondrial platform for recruitment of pro-apoptotic factors, ultimately leading to apoptosis. In contrast, SUMO2/3-DRP1 gets removed from mitochondria thereby protecting the cells from apoptosis. Hypertrophic stimuli also induce the stabilization of PML nuclear bodies (PML-NB) leading to p53 activation and subsequent apoptosis, accompanied, on the other hand, by TGF-β release and cardiac fibrosis. In contrast, RNF4, the SUMO-targeted E3 ubiquitin ligase exerts a protective effect through ubiquitin-mediated degradation of PML or other polySUMOylated targets. In cardiac ischemia/reperfusion (I/R) injury, SENP1 deSUMOylates and stabilizes Hif-1α, an important protective factor promoting cardiomyocyte survival. SENP2 removes SUMO2/3 from Akt rendering the Akt pathway less active in cardiomyocytes. Thus, SENP2 inhibition promotes cardiac de-differentiation, proliferation and angiogenesis via the activation of β-catenin, cyclin D1 and VEGF signaling, respectively. The activity of redox-sensitive SENP3 is regulated in I/R but the specific cardiac targets of SENP3 remain to be clarified. Notably, RNF4 is induced by I/R and counteracts apoptosis most likely by affecting the PML-p53 axis. DJ-1 (also known as PARK7), a cytoprotective factor also induced in I/R, shifts the balance toward SUMO2/3-DRP1 via SENP5 inhibition, thereby preventing mitochondrial fission and subsequent apoptosis.

SENP1 seems to protect cardiomyocytes against hypertrophic growth stimuli as indicated by increased expression of SENP1 in hypertrophic and failing hearts of human patients. Via activation of the calcineurin-NFAT3 (nuclear factor of activated T-cells-3) pathway SENP1 has been interconnected to PGC1-α and subsequent mitochondrial gene activation (Figure 3). This gene expression program is a hallmark of the initial compensatory mechanism upon hypertrophy, limiting the glycolytic metabolic switch in the dysfunctional heart (Cai et al., 2015). Remarkably, deregulated mitochondrial biogenesis causes subsequent loss of sarcomeric structure ultimately leading to cardiomyopathy and heart failure. Mechanistically, deSUMOylation of MEF2C, a MADS-box transcription factor via SENP1 increases its transcriptional activity thereby inducing expression of PGC1-α in the heart (Figure 3) (Cai et al., 2015). Therefore, prolonged expression of SENP1 observed in human failing hearts might contribute to cardiac dysfunction by altered mitochondrial function. Indeed, forced expression by injecting rAAV9-SENP1 in adult hearts induces dilated cardiomyopathy and mitochondrial abnormalities in mice (Cai et al., 2015). However, it remains unclear, whether the observed cardiac phenotype of SENP1 overexpression is fully ascribed to PGC1-α activation. Indeed, SERCA2a, the sarco-/endoplasmic Ca^2+^ATPase might be a good candidate for SENP1 action in heart failure, further deteriorating cardiac contractility by deSUMOylation and subsequent inactivation and degradation of SERCA2a (Figure 3) (Kho et al., 2011).

The importance of a balanced SUMO deconjugation machinery in the adult heart is further supported by cardiac specific overexpression of SENP2, also leading to cardiomyopathy and cardiac dysfunction (Kim et al., 2012). Of note, a subgroup of *a*-myosin heavy chain-SENP2-transgenic mice does not develop septal defects, but shows cardiac hypertrophy and fibrosis during ageing. Since SUMO1 overexpression does rescue the embryonic but not the adult heart phenotype of *a*-myosin heavy chain-SENP2-transgenic mouse (Kim et al., 2012; Kim et al., 2015b), it is possible that SENP2-driven deconjugation of SUMO2/3 is responsible for the adult phenotype. Indeed, by injecting rAAV9-shSENP2 into adult hearts (Chen et al., 2021), knockdown of SENP2 leads to enhanced SUMO2/3ylation of Akt in cardiomyocytes thereby increasing Akt activity, which in turn reduces GSK-3ß levels (Figure 3). Inhibition of GSK-3ß was shown to induce dedifferentiation and proliferation of cardiomyocytes and angiogenesis via increased protein levels of ß-catenin, cyclin D1 and VEGF, respectively (Chen et al., 2021). These findings are consistent with the observed phenotype of the SENP2 knockdown mice characterized by dedifferentiated and hypertrophic cardiomyocytes (Chen et al., 2021). However, it is likely that Akt is not the only target of SENP2 in adult heart responsible for phenotypic changes, leaving a multitude of possible SENP2 targets in the darkness and thereby plenty of room for further investigation.

Cardiac specific overexpression of SENP5 has been linked to mitochondrial fission, which is assumed to rely on enhanced recruitment to mitochondria and oligomerization of the dynamin-related protein 1 (DRP1) (Wasiak et al., 2007; Zunino et al., 2007; Zunino et al., 2009). It has been proposed that SUMO1 conjugation enhances retention of DRP1 on the membrane after recruitment of DRP1 to mitochondria, followed by disassembly of the DRP1 oligomer via de-SUMOylation once fission is complete (Wasiak et al., 2007; Zunino et al., 2007; Zunino et al., 2009). SENP5, SENP3 and SENP2 were all reported to function as deSUMOylating enzymes of DRP1 (Figure 3) (Zunino et al., 2007; Braschi et al., 2009; Zunino et al., 2009). SUMOylation of DRP1 stabilizes an endoplasmic reticulum/mitochondrial platform, which is required for apoptosis (Prudent et al., 2015). In contrast, lack of SENP5 traps SUMO1-conjugated DRP1 at the mitochondrial membrane, ultimately leading to apoptotic cell death. Cardiac specific forced SENP5 expression decreases the level of SUMO2/3 conjugated DRP1 and induces thereby elevated apoptotic cell death, leading ultimately to cardiomyopathy in adult mice (Figure 3). Different SUMO paralogues might fulfil different roles when conjugated to DRP1. It has been proposed that SUMO2/3-DRP1 might prevent its association with mitochondria and cell death (Kim et al., 2015b), whereas SUMO1-DRP1, in contrast, might promote its mitochondrial localization and drive apoptosis (Prudent et al., 2015). Consistent with these findings, SENP3 deconjugates SUMO2/3 from DRP1 in an *in vitro* model of neuronal ischemia reperfusion injury causing apoptotic cell death (Anderson and Blackstone, 2013; Guo et al., 2013). Whether the SENP3/DRP1 axis is relevant in the heart remains to be clarified since unbalanced SUMO conjugation or deconjugation critically depends on the cell type, as well as the physiological or pathological context (Figure 3). In addition, whether SENP3 and SENP5 share common protein targets and exert redundant functions in deSUMOylation or are mainly responsible for distinct sets of targets is still an open question in the field.

Recently, great efforts have been taken to investigate the target specificity of SENP6 (Wagner et al., 2019), though the role of the polySUMO-chain editing enzymes SENP6 and SENP7 in the heart remains unclear, despite the fact that SENP6 and SENP7 are the most highly expressed SENP family members in the human heart throughout the different anatomical regions (Figures 2B,C). Notably, SENP6 and SENP7 play a pivotal role in deSUMOylating the promyelocytic leukemia protein (PML) essential in the assembly of PML nuclear bodies (NB) thereby regulating NB dynamics and diverse cellular functions (Mukhopadhyay et al., 2006; Hattersley et al., 2011; Wagner et al., 2019). It has recently been shown (Liu et al., 2017) that arsenic trioxide injection, known to induce PML-SUMOylation and NB formation, enhanced cardiac fibrosis via upregulation of TGFß1, whereas inhibition of SUMOylated PML by silencing Ubc9, the unique SUMO E2-conjugating enzyme, reduced the development of cardiac fibrosis in mice undergoing transverse aortic constriction (TAC) (Figure 3). In contrast, enhancing SUMOylated PML accumulation, by silencing RNF4, a SUMO targeted E3 ubiquitin ligase (StUbL), accelerated cardiac fibrosis and worsened cardiac function. These findings underline the critical role of Ubc9/PML/RNF4 axis in the pathomechanism of cardiac fibrosis and raise the possibility that SENP6 and SENP7, by counteracting RNF4 in PML SUMOylation (Figure 3), also contribute to this process providing potentially new therapeutic targets for the treatment of cardiac fibrosis and heart failure.

### Acute Coronary Occlusion and Ischemia Reperfusion Injury

Interruption of blood supply to the heart is a leading cause of morbidity and mortality in the western countries (Murray and Lopez, 1997). To limit infarct size and reduce cardiac damage timely reperfusion strategy is routinely applied inducing further ROS-dependent oxidative damage and cardiomyocyte death (Heusch, 2020). Understanding the underlying molecular events is instrumental to find reasonable approaches for cardioprotection by preventing or reversing pathological adaption. Strikingly, in hibernating animals, which provide a model of natural tolerance to ischemia, massive SUMOylation (mainly SUMO2/3) is observed in brain (Sang et al., 2006; Lee et al., 2007). Indeed, the neuroprotective role of enhanced global SUMO2/3ylation have been proved in several cerebral I/R models (Lee and Hallenbeck, 2013; Guo and Henley, 2014; Yang et al., 2014). This raised the obvious question whether inactivation of SENPs is also responsible for shifting the balance toward SUMO conjugation and thereby contributing to cytoprotection. Surprisingly, whereas degradation of the redox-sensitive SENP3 by unfolded protein response (UPS) indeed induced neuroprotection in a DRP1-dependent manner during hypoxia (Yan et al., 2010), SENP1, another redox-sensitive member of the SENPs (Xu et al., 2008) proved to be deleterious upon inactivation (Zhang et al., 2016). It seems likely that SUMOylation by SUMO1, and consequently SUMO1 deconjugation by SENP1 plays a different role during I/R injury than SUMO2/3 deconjugation by SENP3 suggesting distinct SUMO1- versus SUMO2/3-dependent subset of targets and regulatory pathways in the brain.

Regarding the heart, several research groups analyzed the role of the SUMO system in different cellular and animal models of I/R including genetic mouse models of altered SENP1, SENP2 and SENP3 expression (Gu et al., 2014; Mendler et al., 2016; Shimizu et al., 2016; Zhang et al., 2016; Gao et al., 2018; Zhang et al., 2018; Bian et al., 2019; Rawlings et al., 2019; Hotz et al., 2020; Chen et al., 2021)). Depending on the experimental model used, different SUMOylation patterns, SENP alterations and functional outcomes have been observed**.** Since SENP1, -2 and -3 germline deletions cause embryonic lethality, heterozygous knockout mice or rAAV- treated adult wild type (WT) mice (with either SENP knockdown or overexpression) have been used in animal experiments. Regarding SENP1, I/R injury in human and WT murine hearts as well as in isolated cardiomyocytes leads to increased expression (Gu et al., 2014). In addition, heterozygous SENP1 ± mice develop larger myocardial infarct lesion than control animals, altogether indicating that SENP1 might act protective against myocardial I/R injury similar to what was observed in the brain (Figure 3). Because SENP1-mediated deSUMOylation stabilizes HIF1-α under hypoxic conditions (Cheng et al., 2007) and elevated HIF-1α protects against cardiac I/R injury, deSUMOylation of HIF-1α by SENP1 seems to be involved in cardioprotection (Figure 3). Interestingly, knockout of DJ-1 (also known as PARK7), a known cytoprotective factor enhanced accumulation of SUMO1 modified proteins and reduced SUMO2/3 modified proteins in the heart after I/R injury (Shimizu et al., 2016). This was accompanied with reduced SENP1 and higher SENP5 protein expression, leading to enhanced SUMO1- and reduced SUMO2/3 modification of DRP1 with excessive mitochondrial fission as discussed above, worsening the outcome of I/R injury (Figure 3). Although timely controlled and finely balanced SUMO status of specific substrates (but not bulk conjugation/deconjugation) seems to be critical, little is known about distinct proteins targeted by SENP1 in the heart in response to I/R injury (Figure 3).

The role of SENP3 in cardiac I/R injury is more controversial. In agreement with the role of SENP3 in the brain, Gao et al. (2018) reported ROS dependent overexpression of SENP3 in mouse hearts during reperfusion. By examining adult mouse hearts with SENP3 knockdown or overexpression, SENP3 greatly contributed to cardiac I/R injury after LAD (left anterior descending coronary artery) ligation. Notably, other research groups found the opposite, showing protective role for SENP3 in I/R injury in H9C2 cells (Zhang et al., 2018; Rawlings et al., 2019) or during global ischemia and reperfusion in *ex vivo* rat hearts subjected to Langerhans-perfusion (Rawlings et al., 2019). Similarly, SENP3 expression levels varied greatly in different studies during ischemia and reperfusion with increased or unchanged levels following ischemia and reperfusion. Interestingly, Rawlings et al. (2019) observed significant reduction of the cytoplasmic fraction of SENP3 during ischemia paralleled by remarkable increase in nuclear fraction pointing toward a possible relocation of SENP3 to the nucleus (Figure 3). Since none of the studies measured SENP3 enzyme activities, it remained unclear whether increased global or nuclear SENP3 protein levels during I/R correlate with changes in activity. According to our experiments with activity-based assays in wild type mouse heart extracts during ischemia and reperfusion, both SUMO1- and SUMO2/3 dependent SENP activities decreased, with SENP3 being responsible for most changes in SUMO2/3 deconjugation (Hotz et al., 2020) (Figure 3). Accordingly, by performing large-scale SUMO immunoprecipitation, we detected dynamic alterations in several potential SUMO1- and SUMO2/3 cardiac target proteins with proteasomal subunits, chaperones, metabolic enzymes and hypoxia-related transcription factors among them (Hotz et al., 2020). However, further investigations are required to define which of these, mainly SUMO2/3-conjugated proteins are specifically targeted by SENP3. Interestingly, a recent publication revealed a ROS-dependent overexpression and an important role of SENP3 in vascular remodeling via deSUMOylation of ß-catenin and regulation of its stability (Cai et al., 2021). Altogether, SENP3 expression and enzymatic activity seem to undergo a dynamic, organ- and cell-type specific multi-level regulation in ischemia and reperfusion injury. Obviously, the different cellular versus animal I/R models, the severity and time scale of ischemia and reperfusion as well as different protein isolation methods critically influence the experimental outcomes. This again underlines the need to establish and analyze cardiac-specific SENP3 modified mice using standardized protocols to be able to carefully explore cardiac-specific protective or destructive functions of SENP3. To note, SENPs are not only responsible for SUMO deconjugation, but also for processing of SUMO precursors (Figure 1, Nayak and Müller, 2014), therefore, depletion of a given SENP does not necessarily lead to enhanced conjugation in all cases and the consequences on SUMO modification of a given substrate must always be experimentally validated.

Based on proteome data, SENP2 reaches high steady state protein levels among SENPs in the human heart (mainly in left ventricular CM) and seems to undergo less dynamic regulation in the adult heart (Figure 2D, Doll et al., 2017). Interestingly, a recent study reveals that SENP2 deficiency promotes postnatal and adult CM dedifferentiation and proliferation *in vitro* and *in vivo* (Chen et al., 2021)*.* Postnatal increase of SENP2 expression seems to play an important role in the inhibition of CM proliferation since mice with SENP2 deficiency exhibit improved cardiac function after myocardial infarction due to increased CM proliferation and angiogenesis (Figure 3). As discussed earlier, the most striking finding of the study is that SENP2 interacts with and thereby regulates the level of SUMO2/3-Akt. Inhibition of SENP2-mediated Akt deSUMOylation promotes cardiac regeneration via activating Akt pathway, potentially providing a theoretical basis for exiting new therapeutic approaches in cardiac repair after myocardial ischemia. Moreover, since SENP2 plays a significant role in the pathomechanism of atherosclerosis by regulating disturbed-flow induced SUMOylation of ERK5 and p53 leading to endothelial dysfunction, SENP2 might serve as potential target combating atherosclerosis as well (Heo et al., 2013; Heo et al., 2015; Heo et al., 2016; Shetty et al., 2020).

Unfortunately, less is known about the role of SENP6 or SENP7 in cardiac I/R injury. However, an interesting new aspect emerges based on a recent study (Qiu et al., 2020) regarding the role of RNF4 that targets poly-SUMO-modified proteins for ubiquitin-mediated proteolysis (Plechanovová et al., 2011). As discussed above, RNF4 has been implicated in the degradation of PML, a scaffold for multiprotein complex nuclear bodies. Here, knockdown of endogenous RNF4 in adult murine hearts exacerbated oxidative stress-induced cardiomyocyte apoptosis and ischaemia-induced cardiac dysfunction, which was associated with enhanced PML nuclear bodies accumulation, p53 recruitment and activation (Figure 3) (Qiu et al., 2020). We propose that SENP6 and SENP7, by counteracting RNF4 action on PML and, possibly other targets might also interplay in the complex regulatory landscape of ischemic adaptation. Indeed, based on proteomic analysis of different cell lines we and others showed that SENP6 and RNF4 share several targets, with the PIAS family of SUMO E3 ligases among them (Kumar et al., 2017; Keiten-Schmitz et al., 2019; Liebelt et al., 2019; Wagner et al., 2019). Remarkably, PIAS1, the key shared target of SENP6 and RNF4 has been implicated to protect against I/R injury by stimulating PPARγ SUMOylation thereby counteracting the NF-κΒ pathway (Xie et al., 2018). Since PIAS1 was previously shown to regulate several cardiac specific transcription factors (e.g. GATA4 and myocardin; Constanzo et al., 2016) in cardiac development, a broader picture emerges regarding the complex role of SENP6/RNF4 in developing and adult heart. Further investigations in cardiac specific SENP6-, SENP7- and RNF4-gene modified animals are certainly needed to define their functional significance in cardiac pathology.

## Concluding Remarks

Keeping the dynamic balance in SUMOylation/deSUMOylation of cellular proteins is a tightly controlled process in the cardiovascular system. Many aspects of the regulation and function of SENPs have been analyzed *in vitro* and *in vivo*, however, several open questions remain regarding their specific role in the heart. Notably, SENP expression occurs in a region-and cell-type specific manner in healthy human heart with much discrepancies between RNA and protein levels pointing toward a significant posttranscriptional regulation. However, to assess functional consequences of dynamic SENP alterations, activity-based assays should be included to standard procedures when analyzing tissues and organs *in vivo*. Moreover, there is a great need for establishing and carefully analyzing cardiac-specific conditional mice with either SENP knockout/knockdown or overexpression since several controversial data emerge in the literature. According to recent data, overexpression of SENPs induce cardiomyopathic phenotype, however, less is known about the effect of SENP gene silencing in the adult heart. Redox sensing SENPs, *i.e.* SENP1 and SENP3 obviously play a role in ROS dependent ischemia reperfusion injury of the heart, and a striking significance emerges for SENP2 in regulating cardiomyocyte proliferation and cardiac regeneration after myocardial infarction. No information is, however, available about the cardiac specific role of polySUMO-specific SENP6 and SENP7, although the SENP6/PML/RNF4 axis play a central role in several cellular stress responses. In addition, only a handful specific SENP targets (HIF-1α, PGC-1α, DRP1, Akt, PML *etc.*) have been identified so far in adult heart. It is indeed a challenging task to identify and analyze endogenous SUMO targets and to establish deconjugation by a distinct SENP *in vivo*. Further improvement of novel mass spectrometry techniques should allow highly sensitive, proteome-wide identification of SUMO targets. By performing large-scale immunoprecipitations from mouse hearts during I/R injury we uncovered dynamic regulation of potential new SUMO targets including proteasomal subunits, chaperones, muscle-specific ubiquitin ligases, hypoxia- or angiogenesis related transcription factors and metabolic enzymes. The obvious question arises how this knowledge can be converted for clinical use. As deregulated SENP expression is observed in multiple human cancer types SENPs have been emerged as attractive targets in anticancer therapy (Kukkula et al., 2021). However, the similar catalytic site and protein structure makes it challenging to design isoform-selective SENP inhibitors, although these could display lower effective doses and higher drug safety, leading to improved therapeutic outcomes (Tokarz and Woźniak, 2021). Nevertheless, peptide nucleic acids (PNA) have recently been used as novel SENP1 inhibitors in an early phase clinical trial (NCT03798587, Kukkula et al., 2021) which might pave the path for further clinical investigations. Certainly, we need to better understand the specific functions of SENPs in the heart to use them as new therapeutic targets in heart disease.
